# Supplementary material for: Causal Relationship between Aging and Anorexia Nervosa: A White-Matter-Microstructure-Mediated Mendelian Randomization Analysis
Source: Biomedicines. 2024 Aug 16;12(8):1874. doi: 10.3390/biomedicines12081874 (PMC11351342; doi:10.3390/biomedicines12081874)
Supplement: Supplementary file 1 [file biomedicines-12-01874-s001.zip › Supplementary Figure/Figure S8/Leaveoneout plot of MR analysis for TL to ubm-b-1662.pdf]

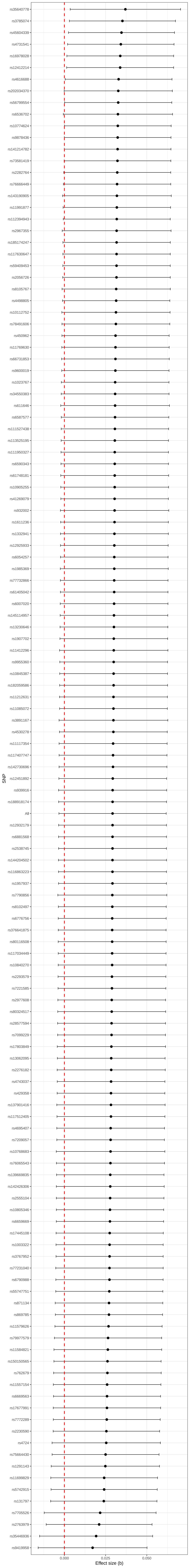
rs35640778
rs3785074
rs45604339
rs4731541
rs16978028
rs12412214
rs4616688
rs202034370
rs56799554
rs6536702
rs10774624
rs9878436
rs141214782
rs73581419
rs2282764
rs76666449
rs143190905
rs11991877
rs112394943
rs2967355
rs185174247
rs117630647
rs59409453
rs2056726
rs8105767
rs4498805
rs10112752
rs78491606
rs450962
rs11769630
rs66731853
rs9600019
rs1023767
rs34550383
rs611646
rs6587577
rs111527438
rs113525195
rs111950327
rs6590343
rs61748181
rs10905255
rs41269079
rs932002
rs1611236
rs1332941
rs12925933
rs6054257
rs1985369
rs77732866
rs61405042
rs6007020
rs145114957
rs13230646
rs1907702
rs11412296
rs9955360
rs10845387
rs182059588
rs12212631
rs11085072
rs3891167
rs4530278
rs11117354
rs117407747
rs142730696
rs12451892
rs939916
rs188918174
All
rs12932179
rs6881568
rs2538745
rs144204502
rs116863223
rs1957937
rs7790856
rs8102497
rs6776756
rs376641875
rs80116508
rs117034449
rs10840270
rs2293579
rs7221585
rs2977608
rs80324517
rs28577594
rs7099229
rs17803849
rs13062095
rs2276182
rs4743037
rs429358
rs137901416
rs117512405
rs4695407
rs7209057
rs10768683
rs76065543
rs139669835
rs142426306
rs2555104
rs10805346
rs6659669
rs17445108
rs1003322
rs3767952
rs77231040
rs6790988
rs55747751
rs871134
rs869785
rs11579626
rs79977579
rs11584821
rs150150565
rs762679
rs11557154
rs6669563
rs17677991
rs7772289
rs2230590
rs4724
rs75664430
rs1291143
rs11699829
rs5742915
rs131797
rs7705526
rs2763979
rs35446936
rs9419958
SNP
0.000
0.025
0.050
Effect size (b)
